# Fitness Dance Counteracts Female Ph.D. Candidates’ Stress by Affecting Emotion Regulation

**DOI:** 10.3390/ijerph192214627

**Published:** 2022-11-08

**Authors:** Datian Liu, Fengxin Sun, Yongsheng Zhu, Changjun Jia, Yupeng Mao, Bing Liu

**Affiliations:** 1 Physical Education Department, Northeastern University, Shenyang 110819, China; 2 School of Arts, Beijing Sport University, Beijing 100084, China

**Keywords:** fitness dance, MBSR, female Ph.D. candidates, stress alleviating, exercise

## Abstract

**Background**: The impact of stress on the nation’s physical and mental health is considerable. Exercise is considered to have beneficial effects on mental health and the capacity for coping with stress. The purpose of this study is to verify the effects of fitness dance intervention on female Ph.D. candidates’ stress, and compare it with the intervention effects of MBSR. **Method**: A repeated measurement experimental design was used to evaluate the effects of fitness dance and MBSR on Chinese female Ph.D. candidates’ stress. **Results**: Twelve weeks of fitness dance and MBSR can reduce participants’ stress from severe to moderate. Eight weeks of fitness dance can reduce the tension from perceived stress (*p* = 0.019) and loss of control from perceived stress (*p* = 0.043). Twelve weeks of fitness dance can reduce the tension from perceived stress (*p* < 0.000), loss of control from perceived stress (*p* = 0.002) and perceived stress (*p* = 0.001). Fitness dance and MBSR affect emotion regulation, thereby affecting stress. Fitness dance reduced participants’ stress by improving their cognitive reappraisal ability. MBSR reduced participants’ stress by improving their cognitive reappraisal ability and expression suppression ability. **Conclusions**: Fitness dance, as a method of exercise intervention, is suitable for reducing Chinese female Ph.D. candidates’ stress.

## 1. Introduction

The impact of stress on the nation’s physical and mental health has been considerable [1,2]. Recent years have focused a considerable amount of public and professional interest on the relationship between stress and physical and mental health. The relationship between stress and health is well documented, with exposure to stressful events linked to depression [3,4], cardiovascular disease [5,6], mortality [1], and numerous other negative affections (e.g., symptoms of anxiety and depression) [7]. Stress is inevitable. Compared with other professions or occupations, sources of stress, anxiety, apprehension, or exhaustion in academia are more prominent [8]. Scholars have classified the stress of Ph.D. candidates into five categories: academic stress, economic stress, social stress, family stress, and daily life stress [9,10]. Among them, the stress of female Ph.D. candidates mainly comes from academic stress and family stress [11]. Previous studies have shown that the academic stress of female Ph.D. candidates mainly comes from doing experiments, writing a doctoral thesis, and publishing doctoral qualification papers [9,10,11]. Furthermore, the family stress of female Ph.D. candidates is related to the role conflict caused by women’s multiple identities [12]. Some studies show that stress also potentiates anxiety and impairs emotion regulation [13,14,15]. The survey by Mao Yupeng et al. (2022) showed that the stress faced by Chinese female Ph.D. candidates made them more anxious [16]. A study by Yukihiro Suzuki et al. (2021) revealed an important mechanism of emotion regulation in response to stress, which reflects the level of modulation induced by a stressor [17]. Many studies show that successful regulation of emotions is essential to cope properly with stress [7,10,17].

Exercise has been considered to have beneficial effects on mental health and the capacity to cope with stress. Indeed, exercise as a moderator of stress levels has been previously investigated in university student populations [18,19]. Moreover, these results suggest an appraisal of exercise in order to reduce psychological distress among students. Aerobic exercise has been thought to influence emotion in a dose-dependent manner [20,21]. Scholar Brown et al. (1988) pointed out that physical exercise as a healthy lifestyle may be a valuable resource to combat stress [22]. Salmon (2001) pointed out that physical exercise can effectively combat anxiety and depression and prevent the adverse effects of stress [23]. Silja et al. (2013) found that physical activity can promote children’s mental health by regulating their neuroendocrine response to stress [24]. Some research results showed that there is a negative correlation between college students’ physical activities and academic stress [25]. Therefore, some scholars believed that students can take part in physical activities as an effective way to manage academic stress [26]. Among them, exercise that is combined with music has a better effect on mental health promotion [27,28,29,30]. Music, with slow or fast rhythm cadences, can control and pace the motions of the body [31]. Engaging with music has benefits for the cognitive, emotional, physiological, and social wellbeing of an individual [32]. Fitness dance is a type of sport that promotes health, advances aesthetic appreciation, and enriches social and cultural life by using gymnastic motions with dance elements with the accompaniment of music. A previous study showed that positive experiences such as vitality, social connection, and positive emotion presented in fitness dance may be important factors to promote mental health [33]. In terms of exercise dose, it has been pointed out that for stressed individuals, moderate-intensity physical activity of more than 30 min three times a week can help alleviate stress [34,35]. In addition, some scholars pointed out that participating in moderate-intensity fitness dance more than twice a week exhibits good compliance [36,37,38]. 

Research on stress relief in the field of psychology mainly focus on mindfulness-related exercises. Mindfulness-related exercises are mainly carried out in groups. Many studies designed mindfulness-based exercise programs as: having a frequency of once per week, an intervention time of generally no less than 4 weeks and no more than 12 weeks, and an exercise time of 1–2.5 h each session [39,40,41]. Cheng K P et al. (2016) conducted a prospective study using a four-week short-term Mindfulness Cognitive Therapy program, and conducted a mindfulness intervention study on 135 medical students at the Malaysian University. The results showed that the students’ perceived stress was significantly reduced [42]. Mindfulness has been shown to mediate the effect of meditation programs on stress in a variety of populations [43,44,45]. In a recent narrative synthesis of studies on the effects of mindfulness on stress in college students, 16 of the 22 studies reported significant decreases in self-reported stress [46]. However, the stress-reducing effects in female Ph.D. candidates is less clear. More research is needed to understand what aspects of mindfulness interventions may be working to reduce stress in female Ph.D. candidates. One of the most popular mindfulness-based interventions is Mindfulness-Based Stress Reduction (MBSR) [47,48]. Research in psychology has supported the use of MBSR to alleviate the anxiety of different groups by reducing stress, managing emotions, and regulating physical and mental states [49,50]. A study found that mindfulness-based intervention is effective for patients with depression and anxiety, besides improving physical health [51]. 

Existing research holds a positive attitude towards the use of fitness dance and MBSR to alleviate stress. The perspective of this study is that fitness dance can be taken as a type of exercise to promote female Ph.D. candidates to develop a healthy lifestyle. This study speculates that in the process of promoting female Ph.D. candidates to develop a healthy lifestyle, the positive emotions generated by participating in fitness dance are conducive to reducing their stress. At the same time, MBSR has been widely used to alleviate stress, but this use has not been confirmed among female Ph.D. candidates. In this paper, the hypothesis is that fitness dance and MBSR affect emotion regulation, thereby affecting stress. To examine this hypothesis, a randomized controlled experiment with fitness dance as the exercise intervention and MBSR as the psychological intervention was designed to explore effects of these two interventions on female Ph.D. candidates’ stress. This study refers to the design of the existing mindfulness-based stress reduction interventions and fitness dance interventions which were used to alleviate stress. The fitness dance intervention was designed as two weekly moderate-intensity sessions of 40 min each. The MBSR intervention was designed as one weekly session of 60 min. They both took place for 12 weeks. The Chinese Perceived Stress Scale was used to investigate the stress of participants. The Emotion Regulation Questionnaire was used to investigate the emotion regulation of participants. Methods, effects, and differences of the two interventions were reported in this study.

## 2. Methods

### 2.1. Participants

Participants were recruited between March and April 2022 via social media and the Northeastern University student affairs office. Interested female Ph.D. candidates were sent the Chinese Perceived Stress Scale (CPSS). Participants were eligible for the study if they were current full-time female Ph.D. candidates and (1) scored ≥14 points on the Chinese Perceived Stress Scale (CPSS), (2) were willing to be randomized, and (3) did not take drugs. Participants were excluded if they had a current fitness dance practice or mindfulness practice. Eighty-five female Ph.D. candidates from Northeastern University were the participants for this study. Their age was 28.43 ± 3.71 years. There were 43 participants in the fitness dance group and 42 participants in the MBSR group. Table 1 is the specific information. This paper follows the following ethical principles in the implementation of the experiment. Before the start of the research, the participants were introduced in detail to the purpose, methods, and possible risks in the process of the research. They were told that they could voluntarily decide whether to participate or not on the basis of fully understanding the research process. They were also informed that they had the right to withdraw freely at any time, and filled in the informed consent form after consent was given. (2) The personal information and research data of the participants are strictly confidential. (3) During the research, we took various measures to ensure the personal safety of the participants. The approval number of this research is “No.:202203-15”.

### 2.2. Instruments

The Chinese Perceived Stress Scale (CPSS) (Yang Tingzhong, China) was used to investigate the stress of Chinese female Ph.D. candidates. The Perceived Stress Scale includes three versions: PSS-14, PSS-10, and PSS-4. Yang Tingzhong et al. first adapted PSS-14 for a Chinese context, and introduced it to China in 2003 [50]. The Cronbach’s alpha of the Chinese version of the PSS (CPSS) is 0.78, and the correlation coefficient between each item and the total score is 0.37–0.53, which means the scale has good credibility. The scale has two dimensions, namely *tension* and *sense of loss of control*, and rates on a five-point Likert scale ranging from 1 (“Never”) to 5 (“Always”). Many studies have shown that the CPSS has good reliability and validity [52,53,54,55]. Given the preestablished psychometric properties of the survey tools, construct validity and reliability diagnostics were not performed for this study.

The Emotion Regulation Questionnaire (ERQ) was used to investigate the emotion regulation of Chinese female Ph.D. candidates. The Emotion Regulation Questionnaire was designed to assess individual differences in the habitual use of two emotion regulation strategies: *cognitive reappraisal* and *expressive suppression* [56]. The ERQ comprises 10 items and rates on a seven-point Likert scale ranging from 1 (“strongly disagree”), to 4 (“neutral”), to 7 (“strongly agree”). Participants were asked to rate the statements according to how they reappraised or suppressed the expression of emotions. The validity, reliability, and factor structure of this questionnaire has been demonstrated [56], and replicated consistently [57,58,59,60].

### 2.3. Procedures

From May to July in 2022, the fitness dance group received two weekly sessions of 40 min of moderate-intensity fitness dance exercise for 12 weeks. The MBSR group received one weekly session of 60 min of MBSR for 12 weeks. Both of the two groups completed the exercise in a group environment. The participants in the fitness dance group were organized in one group to practice, and their course was taught by a female doctor who majored in physical education and training. The participants in the MBSR group were organized into two groups to practice, and their course was taught by a female doctor who majored in positive psychology. Both of the two groups were administered the testing instruments at baseline (before starting the 12-week exercise program), the 8th week, and the 12th week, to assess perceived stress and emotion regulation. The instruments were the Chinese Perceived Stress Scale (CPSS) and Emotion Regulation Questionnaire (ERQ).

### 2.4. Statistical Analysis

SPSS 21.0 statistical software (IBM, Armonk, NY, USA) was used for descriptive statistics, analysis of variance, correlation analysis, and regression analysis.

## 3. Results

### 3.1. Basic Information of Participants

The grouping results showed that there were 43 participants in the fitness dance group and 42 participants in the MBSR group. Chi-square test showed that there was no difference in age, marital status, motherhood, and grade between the two groups (*p* > 0.05, Age = 0.983, Marital status = 0.828, Motherhood = 0.972, Grade = 0.391). The two groups were comparable. Table 1 is the specific information. After interventions, there were 38 participants in the fitness dance group and 38 participants in the MBSR group. The reasons for the loss of nine participants include: poor compliance (5), business trip (1), heavy learning task, and withdrawal from practice (3).

### 3.2. Comparison of Baseline Balance of Perceived Stress and Emotion Regulation between Two Groups of Participants

Table 2 is the baseline perceived stress and emotion regulation of female Ph.D. candidates in the fitness dance group and the MBSR group. The result of variance analysis showed that there was no difference between the two groups in the variables of “CPSS-Tension”, “CPSS-Loss of control”, “CPSS”, “ERQ-Cognitive reappraisal”, “ERQ-Expressive suppression” (*p >* 0.05). The two groups were comparable.

### 3.3. Effects of Fitness Dance and MBSR on Female Ph.D. Candidates’ Perceived Stress and Emotion Regulation

As shown in Table 3, the results showed that the time main effect of “CPSS-Tension” of the two groups at different time points was significant (F = 1.039, *p* = 0.008, η^2^p = 0.829). The main effect of group was significant (F = 0.702, *p* = 0.038, η^2^p = 0.122). The interaction between time and group was significant (F = 0.893, *p* = 0.026, η^2^p = 0.171). Table 4 shows further simple effect analysis found that it was significant at the 8th week (*p* = 0.043) and 12th week (*p* = 0.032).

The time main effect of “CPSS-Loss of control” of the two groups at different time points was significant (F = 2.232, *p* = 0.021, η^2^p = 0.918). The main effect of group was significant (F = 2.013, *p* = 0.027, η^2^p = 0.224). The interaction between time and group was significant (F = 3.965, *p* = 0.005, η^2^p = 0.102). Table 4 shows further simple effect analysis found that it was significant at the 8th week (*p* = 0.018) and the 12th week (*p* = 0.044).

The time main effect of “CPSS” of the two groups at different time points was significant (F = 2.07, *p* = 0.009, η^2^p = 0.829). The main effect of group was not significant (F = 0.002, *p* = 0.528, η^2^p = 0.016). The interaction between time and group was not significant (F = 0.012, *p* = 0.473, η^2^p = 0.006). 

The time main effect of “ERQ-Cognitive reappraisal” of the two groups at different time points was significant (F = 1.019, *p* = 0.042, η^2^p = 0.644). The main effect of group was not significant (F = 0.917, *p* = 0.052, η^2^p = 0.09). The interaction between time and group was not significant (F = 0.537, *p* = 0.067, η^2^p = 0.088).

The time main effect of “ERQ-Expressive suppression” of the two groups at different time points was significant (F = 1.897, *p* = 0.027, η^2^p = 0.425). The main effect of group was not significant (F = 0.521, *p* = 0.732, η^2^p = 0.152). The interaction between time and group was significant (F = 1.02, *p* = 0.041, η^2^p = 0.07). Table 4 shows further simple effect analysis found that it was significant at the 8th week (*p* = 0.034), but it was not significant at the 12th week (*p* = 0.122). 

The results of perceived stress of the two groups at the 8th and 12th week showed (Table 5) that, after 8 weeks of intervention, the participants’ “CPSS-Tension” and “CPSS-Loss of control” in the fitness dance group were significant (*p* = 0.019, *p* = 0.043), but their “CPSS” was not significant (*p* = 0.051). In the MBSR group, the participants’ “CPSS-Loss of control” and “CPSS” were significant (*p <* 0.000, *p <* 0.000), but their “CPSS-Tension” was not significant (*p* = 0.231). After 12 weeks of intervention, the participants’ “CPSS-Tension”, “CPSS-Loss of control”, and “CPSS” in the fitness dance group were significant (*p <* 0.000, *p* = 0.002, *p* = 0.001). In the MBSR group, the participants’ “CPSS-Tension”, “CPSS-Loss of control”, and “CPSS” were also significant (*p* = 0.021, *p <* 0.000, *p <* 0.000). The results of differences between the 8th week and 12th week showed that the participants’ “CPSS-Tension”, “CPSS-Loss of control”, and “CPSS” in the fitness dance group were significant (*p* = 0.008, *p* = 0.01, *p* = 0.012). In the MBSR group, the participants’ “CPSS-Loss of control” and “CPSS” was significant (*p <* 0.000, *p* = 0.009).

The results of emotion regulation of the two groups at the 8th and 12th week showed (Table 5) that, after 8 weeks of intervention, the participants’ “ERQ-Cognitive reappraisal” in the fitness dance group was significant (*p <* 0.000), but their “ERQ-Expressive suppression” was not significant (*p* = 0.102). In the MBSR group, the participants’ “ERQ-Cognitive reappraisal” and “ERQ-Expressive suppression” were both significant (*p <* 0.000, *p* = 0.049). After 12 weeks of intervention, the participants’ “ERQ-Cognitive reappraisal” in the fitness dance group was significant (*p <* 0.000), but their “ERQ-Expressive suppression” was not significant (*p* = 0.051). In the MBSR group, the participants’ “ERQ-Cognitive reappraisal” and “ERQ-Expressive suppression” were both significant (*p <* 0.000, *p* = 0.047). The results of differences between the 8th week and 12th week showed that the participants’ “ERQ-Cognitive reappraisal” and “ERQ-Expressive suppression” in the fitness dance group were significant (*p* = 0.006, *p* = 0.047). In the MBSR group, the participants’ “ERQ-Cognitive reappraisal” was significant (*p* = 0.002).

### 3.4. Correlation between Perceived Stress and Emotion Regulation in the Two Groups

As shown in Table 6, the results showed that perceived stress in the two groups (the fitness dance group and the MBSR group) was positively correlated with grade, CPSS-tension, CPSS-loss of control, ERQ-expressive suppression, and negatively correlated with ERQ cognitive reappraisal.

Based on the correlation analysis, perceived stress was taken as the dependent variable. The grade, tension, loss of control, cognitive reappraisal and expressive suppression were taken as the independent variables in the stepwise regression analysis. The results of the fitness dance group (Table 7) showed that the grade did not affect perceived stress at the 8th and 12th week. The tension and loss of control significantly positively predicted the score of perceived stress at the 8th and 12th week. The cognitive reappraisal significantly negatively predicted the score of perceived stress at the 8th and 12th week. The expressive suppression positively predicted the score of perceived stress at the 8th week. 

The results of the MBSR group (Table 8) showed that the grade positively predicted perceived stress at the 8th week. The tension, loss of control, and expressive suppression significantly positively predicted the score of perceived stress at the 8th and 12th week. The cognitive reappraisal negatively predicted the score of perceived stress at the 8th and 12th week.

## 4. Discussion

This study showed that fitness dance and MBSR can reduce Chinese female Ph.D. candidates’ stress from severe to moderate. Specifically, fitness dance alleviated stress by reducing the tension of participants’ perceived stress and improving their cognitive reappraisal ability. Compared with fitness dance, MBSR mainly reduced the loss of control aspect of perceived stress on participants, and reduced the use of expressive suppression strategies to reduce stress. Cognitive reappraisal involves an attempt to generate a positive interpretation of a traumatizing event to reduce excessive emotionality. Expressive suppression names the attempt to modulate negative emotions by hiding, inhibiting, or reducing the behavioral response to a stressful event. Cognitive reappraisal, but not expressive suppression, is effective in reducing physiological arousal and negative emotions [62,63], and is associated with beneficial physiological and psychological outcomes [64,65]. This study also indicated that within the context of stress, expressive suppression is associated with higher levels of stress.

This study also aimed to compare the effects of fitness dance and MBSR on perceived stress and emotion regulation in Chinese female Ph.D. candidates. From the perspective of intervention time, participants reported that fitness dance reduced the tension score of participants’ perceived stress at the 8th week. MBSR reduced the participants’ sense of loss of control of perceived stress at the 8th week. At the 12th week, the two interventions both significantly reduced the total perceived stress score of the participants. From the influence of fitness dance and MBSR on the perceived stress of Chinese female Ph.D. candidates, fitness dance showed a better function of reducing the tension of perceived stress, and the reduction of perceived stress begins at the 12th week. Compared with fitness dance, MBSR reduced perceived stress at the 8th week, mainly by reducing the sense of losing control attributed to perceived stress of Chinese female Ph.D. candidates.

The important finding of this study was that the emotional regulation produced by fitness dance and MBSR has a certain mediating effect on the relationship between Chinese female Ph.D. candidates and stress. Specifically, fitness dance reduced the perceived stress of participants by improving their cognitive reappraisal ability. MBSR alleviates the perceived stress of participants by improving their cognitive reappraisal ability and expression suppression ability. The initial hypothesis of this study was that fitness dance and MBSR affect emotion regulation, thereby affecting stress, and the results confirmed the primary hypothesis. The perceived stress of the participants in the fitness dance group and the MBSR group was positively correlated with expressive suppression and negatively correlated with cognitive reappraisal. The following analysis of MBSR and fitness dance functions may provide preliminary support for the results. Existing studies have consistently shown that MBSR is a kind of psychological education, and its impact on individual cognitive reappraisal strategies and expression suppression strategies is conducive to reducing stress [56,66,67]. A systematic review with a meta-analysis indicated that psychoeducation is effective in both reducing stress and in helping people to manage stress due to trauma [68]. Fitness dance is a form of exercise intervention. The literature suggests that square dance intervention [69], aerobic dance [70], and African dance [71] are effective in reducing stress, depression, and anxiety. The findings of this study provided preliminary support for the view that fitness dance possibly reduces stress through a mechanism different from that employed in MBSR. The music contained in fitness dance has the ability to regulate emotional state and promote physical and mental interaction, and has been widely used for relaxation in medical care [72,73]. The motions of fitness dance make body and spirit perceive each other, which is the process of sensory information inside the body being transmitted and communicated to the brain and other body structures that occurs with or without conscious attention [74]. Interoception acts as a distractor to shift the individual’s attention away from the negative emotions and replace them with positive thoughts [75]. This shifting of attention serves as one of the emotion-focused coping strategies allowing individuals to cope with stress. As a result, stress and negative emotions could be healed [75]. Moreover, fitness dance is a collective sport. Research supporting the physical and mental health contributions of exercise has identified mediators such as organizational practices and the role of seeing other people who are similar to you becoming and being active, which are significant determinants of exercise engagement [76]. The evidence by Louise Mansfield et al. (2018) suggested that peer-supported delivery mechanisms in sport and dance programs may support wellbeing enhancement for young people [77]. In addition, a previous study showed that the “vitality”, “social connection”, “positive emotion”, and other positive subjective experiences contained in fitness dance are related to the improvement of cognitive reappraisal ability [33]. According to emotion regulation theory, emotion regulation can be controlled consciously and diligently, or it can be carried out automatically without consciousness and effort [61]. In the process of practicing fitness dance, female Ph.D. candidates pay attention to fitness dance practice. Many studies have shown that transference of attention can effectively regulate emotions [78,79,80], reduce stress, and reduce the level of anxiety [81,82,83]. Furthermore, fitness dance’s elements of music and body motion stimulate interoception and act as distractors to shift the individual’s attention away from negative emotions and stress and replace them with positive thoughts [7,84,85]. In the research on exercise intervention similar to fitness dance, Gao et al. (2016) showed that square dance intervention for 3 months could significantly reduce the depressive symptoms of perimenopausal women [69]. Studies pointed out that aerobic dance is popular among middle-aged women because it can help reduce stress, depression, and anxiety [70,86,87]. 

The results also indicated that the stress of female Chinese Ph.D. candidates is related to their grades. Performing experiments, writing a doctoral thesis, and publishing doctoral qualification papers require considerable time, energy, and financial resources [12]. With the increase of grades, if Ph.D. candidates want to graduate on time, they will have less time to complete their studies and more stress in the face of graduation and employment. As such, grade positively affects the stress score of female Chinese Ph.D. candidates.

Although the findings in this study are promising regarding the efficacy of fitness dance and MBSR interventions to reduce stress among Chinese female Ph.D. candidates, several limitations must be noted. First, the sample comprised Chinese female Ph.D. candidates; therefore, the generalizability of our findings may be limited. In future interventions, it is important to explore efficacy in more diverse samples. Second, all data collected in this study relied on self-report, which may lead to social desirability or response bias where participants respond in what they believe is a more favorable manner. Future studies warrant determining the best control condition.

## 5. Conclusions

The findings in this study showed that 12 weeks of fitness dance and MBSR can reduce Chinese female Ph.D. candidates’ stress from severe to moderate. Fitness dance mainly reduces Chinese female Ph.D. candidates’ stress by improving their cognitive reappraisal ability of emotion regulation. As a type of exercise intervention, fitness dance is suitable for reducing Chinese female Ph.D. candidates’ stress. MBSR, as has been shown in previous studies, can reduce Chinese female Ph.D. candidates’ stress by improving their cognitive reappraisal ability and expression suppression ability of emotion regulation in short period of time (8 weeks). It is worth noting that grade has an impact on female Ph.D. candidates’ perceived stress, which should be included in the analysis of variables in future research. This study indicates that fitness dance, as a method of exercise intervention, is suitable for reducing Chinese female Ph.D. candidates’ stress. Future research should be expanded to combine fitness dance with psychological education, such as MBSR and CBT (Cognitive Behavioral Therapy).

This study enlightens us that exercise intervention and psychological intervention are both ways to counteract stress. They are the methods aimed at promoting a healthy lifestyle. Intervention programs to relieve stress for different people should be formulated according to their physical and mental characteristics and sources of stress.

## Figures and Tables

**Table 1:** Comparison of demographic data balance between two groups of female Ph.D. candidates (*n* = 85).

Essential Information	Classification Criteria	Frequency (*n* = 85)	Fitness Dance Group (*n* = 43)	MBSR (*n* = 42)	χ^2^	*p*
Age	20–25 years old	9	4 (9.30%)	5 (11.91%)	0.162	0.983
	26–30 years old	51	28 (65.11%)	23 (54.76%)		
	31–35 years old	23	10 (23.26%)	13 (30.95%)		
	36–40 years old	2	1 (2.33%)	1 (2.38%)		
Marital status	Unmarried	68	37 (86.04%)	31 (73.81%)	0.647	0.828
	Married	17	6 (13.96%)	11 (26.19%)		
Motherhood	No	77	41 (95.34%)	36 (85.71%)	0.531	0.972
	Yes	8	2 (4.66%)	6 (14.29%)		
Grade	Grade 1	19	10 (23.26%)	9 (21.43%)	0.041	0.391
	Grade 2	22	11 (25.58%)	11 (26.19%)		
	Grade 3	24	12 (27.91%)	12 (28.57%)		
	Grade 4	20	10 (23.26%)	10 (23.81%)		

χ^2^: Variation level between the two groups of samples. *p*: Significance level of samples’ variation between the two groups.

**Table 2:** Comparison of baseline balance of perceived stress and emotion regulation between two groups of participants (M ± SD).

Variable	Fitness Dance Group (*n* = 43)	MBSR (*n* = 42)	F	*p*
CPSS-Tension	24.84 ± 2.05	24.29 ± 1.47	2.028	0.158
CPSS-Loss of control	28.23 ± 1.76	28.33 ± 1.95	0.063	0.803
CPSS	53.07 ± 2.89	52.62 ± 2.69	0.554	0.459
ERQ-Cognitive reappraisal	19.00 ± 3.14	18.90 ± 3.40	0.018	0.894
ERQ-Expressive suppression	18.37 ± 2.57	17.83 ± 2.76	0.868	0.354

F: The ratio of samples’ variance between the two groups. *p*: Significance level of samples’ variance between the two groups.

**Table 3:** Comparison of perceived stress and emotion regulation changes between the two groups before and after intervention (M ± SD).

		CPSS-Tension	CPSS-Loss of Control	CPSS	ERQ-Cognitive Reappraisal	ERQ-Expressive Suppression
Fitness dance group (*n* = 38)	Baseline	24.84 ± 2.02	28.34 ± 1.81	53.18 ± 2.99	18.92 ± 3.20	18.34 ± 2.44
	8th week	21.66 ± 1.81	27.32 ± 1.32	48.97 ± 2.35	22.47 ± 2.63	17.61 ± 1.73
	12th week	18.05 ± 1.33	22.66 ± 1.32	40.71 ± 2.04	24.92 ± 2.36	16.05 ± 1.89
MBSR group (*n* = 38)	Baseline	24.26 ± 1.45	28.21 ± 1.89	52.47 ± 2.60	18.95 ± 3.51	17.74 ± 2.87
	8th week	23.18 ± 1.59	25.61 ± 1.28	48.79 ± 2.13	21.71 ± 3.00	15.42 ± 2.04
	12th week	19.53 ± 1.20	21.00 ± 2.14	40.53 ± 2.64	23.37 ± 2.79	14.87 ± 1.89
Time	F	1.039	2.232	2.07	1.019	1.897
	*p*	0.008 **	0.021 *	0.009 **	0.042 *	0.027 *
	η^2^p	0.829	0.862	0.918	0.644	0.425
Group	F	0.702	2.013	0.002	0.917	0.521
	*p*	0.038 *	0.027 *	0.528	0.052	0.732
	η^2^p	0.122	0.224	0.016	0.09	0.152
Time × Group	F	0.893	3.965	0.012	0.537	1.02
	*p*	0.026 *	0.005 **	0.473	0.067	0.041 *
	η^2^p	0.171	0.102	0.006	0.088	0.07

* *p* < 0.05, ** *p* < 0.01. F: The ratio of samples’ variance between the two groups. *p*: Significance level of samples’ variance between the two groups. η^2^p: Effect size. 0.04 = small, 0.25 = medium, 0.64 = large [61].

**Table 4:** The simple effect analysis of Time× Group of “Fitness dance—MBSR”.

Fitness Dance—MBSR	CPSS-Tension		CPSS-Loss of Control		ERQ-Expressive Suppression	
	t	*p*	t	*p*	t	*p*
8th week	−5.695	0.043 *	7.976	0.018 *	7.853	0.034 *
12th week	−7.121	0.032 *	6.151	0.044 *	2.583	0.122

* *p* < 0.05.

**Table 5:** Comparison of perceived stress and emotion regulation between the two groups at the 8th and 12th week.

Group	Variable	Baseline–8th Week		Baseline–12th Week		8th Week–12th Week	
		t	*p*	t	*p*	t	*p*
Fitness dance group (*n* = 38)	CPSS-Tension	−2.461	0.019 *	−6.151	<0.000 **	−3.882	0.008 **
	CPSS-Loss of control	−1.868	0.043 *	−4.199	0.002 **	−3.395	0.01 *
	CPSS	−1.065	0.051	−4.478	0.001 **	−3.219	0.012 *
	ERQ-Cognitive reappraisal	6.823	<0.000 **	7.26	<0.000 **	5.329	0.006 **
	ERQ-Expressive suppression	−0.504	0.102	−1.19	0.051	−1.69	0.047 *
MBSR group (*n* = 38)	CPSS-Tension	−0.892	0.231	−2.081	0.021 *	−1.036	0.68
	CPSS-Loss of control	−5.337	<0.000 **	−6.833	<0.000 **	−5.916	<0.000 **
	CPSS	−5.05	<0.000 **	−6.27	<0.000 **	−3.785	0.009 **
	ERQ-Cognitive reappraisal	5.629	<0.000 **	5.684	<0.000 **	4.15	0.002 **
	ERQ-Expressive suppression	−1.279	0.049 *	−1.71	0.047 *	−0.029	0.254

* *p* < 0.05, ** *p* < 0.01. t: Variation within group. *p*: Significance level of samples’ variance between the two groups.

**Table 6:** Correlation analysis of perceived stress and emotion regulation in the two groups.

Group	Variable	CPSS		
		Baseline	8th Week	12th Week
Fitness dance group (*n* = 38)	Grade	0.126 *	0.076	0.045
	CPSS-Tension	0.669 **	0.919 **	0.696 **
	CPSS-Loss of control	0.504 **	0.372 *	0.108 *
	ERQ-Cognitive reappraisal	0.998 **	0.606 **	0.402 **
	ERQ-Expressive suppression	0.916 **	0.770 *	0.307
MBSR group (*n* = 38)	Grade	0.134 *	0.105 *	0.029
	CPSS-Tension	0.742 **	0.515 *	0.334 *
	CPSS-Loss of control	0.559 **	0.642 **	0.617 **
	ERQ-Cognitive reappraisal	0.954 **	0.656 **	0.967 **
	ERQ-Expressive suppression	0.992 **	0.825 **	0.513 **

* *p* < 0.05, ** *p* < 0.01.

**Table 7:** Stepwise regression analysis results of fitness dance group.

Variable	Baseline	8th Week	12th Week
Grade	0.786 * (0.923)	—	—
CPSS-Tension	2.543 ** (2.310)	1.093 ** (1.575)	1.855 ** (1.083)
CPSS-Loss of control	1.087 * (7.394)	0.389 * (3.614)	0.056 * (0.768)
ERQ-Cognitive reappraisal	−5.654 ** (−3.276)	−9.011 ** (−0.190)	−3.865 * (−0.098)
ERQ-Expressive suppression	0.647 * (0.812)	0.753 * (0.967)	—
R^2^	0.821	0.886	0.601
Adjusted R^2^	0.818	0.880	0.578
F	27.824 **	36.625 **	26.349 **

Dependent variable: Perceived stress, * *p* < 0.05, ** *p* < 0.01.

**Table 8:** Stepwise regression analysis results of MBSR group.

Variable	Baseline	8th Week	12th Week
Grade	0.886 * (1.023)	0.629 * (0.574)	—
CPSS-Tension	2.675 ** (2.468)	0.859 * (1.293)	1.039 * (0.765)
CPSS-Loss of control	1.001 * (7.165)	5.674 ** (3.574)	4.267 ** (2.765)
ERQ-Cognitive reappraisal	−5.473 ** (−3.126)	−7.246 ** (−5.825)	−0.846 * (−1.506)
ERQ-Expressive suppression	0.573 ** (0.801)	2.708 ** (3.812)	3.676 ** (5.670)
R^2^	0.768	0.765	0.945
Adjusted R^2^	0.761	0.745	0.942
F	34.786 **	36.966 **	30.892 **

Dependent variable: Perceived stress, * *p* < 0.05, ** *p* < 0.01.

## Data Availability

The data presented in this study are available on request from the corresponding author.
